# New Early Warning Score: EMS Off-Label Use in Out-of-Hospital Patients

**DOI:** 10.3390/jcm10122617

**Published:** 2021-06-14

**Authors:** Federico Semeraro, Giovanni Corona, Tommaso Scquizzato, Lorenzo Gamberini, Anna Valentini, Marco Tartaglione, Andrea Scapigliati, Giuseppe Ristagno, Carmela Martella, Carlo Descovich, Cosimo Picoco, Giovanni Gordini

**Affiliations:** 1 Department of Anaesthesia, Intensive Care and Emergency Medical Services, Ospedale Maggiore, 40133 Bologna, Italy; gambero6891@hotmail.it (L.G.); mrc.tartaglione@gmail.com (M.T.); C.Picoco@118er.it (C.P.); giovanni.gordini@ausl.bologna.it (G.G.); 2 Endocrinology Unit, Maggiore-Bellaria Hospital, 3-40139 Bologna, Italy; jocorona@libero.it; 3 Department of Anaesthesia and Intensive Care, IRCCS San Raffaele Scientific Institute, 20132 Milan, Italy; tscquizzato@gmail.com; 4 School of Medicine, Vita-Salute San Raffaele University, 20132 Milan, Italy; 5 Department of Medical and Surgical Sciences, Alma Mater Studiorum University, 40126 Bologna, Italy; annaval.312@gmail.com (A.V.); carmela.martella83@gmail.com (C.M.); 6 Institute of Anaesthesia and Intensive Care, Catholic University of the Sacred Heart, Fondazione Policlinico Universitario A. Gemelli, IRCCS, 00168 Rome, Italy; andrea.scapigliati@gmail.com; 7 Department of Pathophysiology and Transplantation, University of Milan, 00168 Milan, Italy; gristag@gmail.com; 8 Department of Anesthesiology, Intensive Care and Emergency, Fondazione IRCCS Ca’ GrandaOspedale Maggiore Policlinico, 20122 Milan, Italy; 9 Clinical Governance and Quality Unit, Bologna Local Healthcare Authority Staff, 40133 Bologna, Italy; carlo.descovich@ausl.bologna.it

**Keywords:** cardiac arrest, EMS, National Early Warning Score, NEWS, ambulance, apps, out-of-hospital

## Abstract

Background: The National Early Warning Score (NEWS) is an assessment scale of in-hospital patients’ conditions. The purpose of this study was to assess the appropriateness of a potential off-label use of NEWS by the emergency medical system (EMS) to facilitate the identification of critical patients and to trigger appropriate care in the pre-hospital setting. Methods: A single centre, longitudinal, prospective study was carried out between July and August 2020 in the EMS service of Bologna. Home patients with age ≥ 18 years old were included in the study. The exclusion criterion was the impossibility to collect all the parameters needed to measure NEWS. Results: A total of 654 patients were enrolled in the study. The recorded NEWS values increased along with the severity of dispatch priority code, the EMS return code, the emergency department triage code, and with patients’ age (r = 0.135; *p* = 0.001). The aggregated value of NEWS was associated with an increased risk of hospitalization (OR = 1.30 (1.17; 1.34); *p* < 0.0001). Conclusion: This study showed that the use of NEWS in the urgent and emergency care services can help patient assessment while not affecting EMS crew operation and might assist decision making in terms of severity-code assignment and resources utilization.

## 1. Introduction

Early identification of clinical deterioration has proven to improve outcomes in the treatment of acute illnesses [1]. Especially in the pre-hospital emergency system, it is essential to guarantee appropriate assistance through an efficient coordination of the emergency team. Recently, based on a systematic review, the International Liaison Committee on Resuscitation (ILCOR) suggested that hospitals should consider the introduction of a rapid response system (rapid response team/medical emergency team) to reduce the incidence of in-hospital cardiac arrest and in-hospital mortality [2]. Several Early Warning Scores (EWS) have been carried out in different in-hospital settings, each one based on the concept of the track and trigger system (TTS), an approach built on the detection of abnormalities in the main vital signs in order to predict the occurrence of acute adverse events [3]. The EWS is included in the aggregate weighted TTS (AWTTS), in which points are allocated in a weighted manner according to the derangement of variables in the patients’ vital signs from a considered normal range. The sum of the allocated points is known as the Early Warning Score (EWS). Since multiple and different EWS were used within the British National Health Service and worldwide, in 2012, the Royal College of Physicians (RCP) introduced the National Early Warning Score (NEWS) to standardize the approach nationwide [4] (Appendix A: example of table with the score structure).

The NEWS variables for which points are allocated in the score include respiratory rate, oxygen saturation, temperature, systolic blood pressure, heart rate, level of consciousness, plus an additional two points added for any patient requiring supplemental oxygen. Based on the severity of the vital sign, a score ranging from 0 to 3 can be assigned to each parameter. The RCP recommends four trigger levels to alert for requiring clinician assessment. In particular, an aggregate NEWS of 5 or more is a critical threshold that should demand for the call of an urgent clinical evaluation; a NEWS of 7 or more should trigger a high-level clinical alert, i.e., an emergency clinical review. The main aim of NEWS, according to RCP, is to improve the assessment of acute illness severity, the detection of clinical deterioration, and the initiation of a timely and competent clinical response for inpatients. NEWS may help to trigger the most appropriate care in the pre-hospital setting, such as the activation or the denial of the advanced response team; in addition, it may help to choose the appropriate receiving hospital for the patient. Moreover, it could help anticipate the involvement of the senior emergency department or critical-care staff in the receiving hospital. However, its use in the pre-hospital setting is controversial partly due to of lack of evidence. This study aims to investigate whether the pre-hospital use of NEWS by the emergency medical system (EMS) can facilitate the identification of critical patients during hospital admission. 

## 2. Materials and Methods

### 2.1. Study Design

This single center, longitudinal, prospective study was approved by the local ethical committee on 22 April 2020, and it was carried out simultaneously on three ambulances of the local EMS. The overall recruitment period of the study was 2 months (July and August 2020). The study included all patients aged ≥18 years old evaluated on the field by the EMS ambulance crews. The only exclusion criterion was the inability of the ambulance personnel to collect all the parameters needed to calculate NEWS. The EMS personnel collected data on all the requested parameters at the first evaluation of the patient. The NEWS data were recorded using forms on a specifically created web app.

### 2.2. Setting

The Emilia Est Emergency Dispatch Centre (EEE-DC) manages the emergency calls and dispatches vehicles for the three provinces of Bologna, Ferrara, and Modena, covering a total of 2.5 million inhabitants. The urban area of Bologna has a population of 450,739 inhabitants. EMS is composed of eight ambulances equipped with BLS-skilled rescuers (BLS vehicles), four ambulances equipped with immediate life-support experienced nurses (ILS vehicles), two medical cars carrying an enhanced critical-care team composed of an emergency physician (emergency medicine or anaesthesia and intensive care specialists) and a nurse with advanced life-support skills (ALS vehicles). Two BLS vehicles and 1 ILS vehicle were included in the study as a representative sample of the overall activities. In our emergency-call-handling chain, the patient was assessed three times by our emergency system, which led to identification of three main severity codes. First of all, to activate the appropriate vehicle, the EEE-DC delivered an EMS dispatch priority code based on emergency call information; the code can be white, green, yellow, or red depending on the severity of patient conditions as evaluated by the dispatch algorithm. Then, the rescuers on the field assign the so-called return code after completion of patient evaluation; it consists of a number ranging from 0 and 4, where zero represents patient who has been treated on scene or refused the transport; a number from one to three stands for a lower severity to a higher one, respectively; and, lastly, number 4 indicates that the patient died. Finally, when in the Emergency Department, a triage code completes the chain, which can be a green, yellow, or red, indicating increasing severity of the clinical conditions.

### 2.3. Materials and Training

A web application dedicated to NEWS collection data was created. It was developed by a team of volunteers during the national COVID-19 lockdown in Italy between March and May 2020 and named CovidUP19 project, since its original purpose was to collect data from patients quarantined at home [5]. The ambulance crew could use the software with every browser directly on smartphones. The data collection form was designed to collect data in an anonymous way and with a user-friendly approach (Appendix B: CovidUP19 web-app screenshot). The training of all the ambulance crews involved in the study was performed with the use of virtual meetings on Teams (Microsoft Redmond, Washington, DC, USA), accordingly with the COVID-19 local rules for training. The crew members were also invited to use the web app on the field in the training period (June 2020) to warm up and get familiar with it. Overall, sixty-nine EMS personnel were trained on the aim of the study, the study protocol, and the rules to measure vital parameters with a standardized approach and to collect data for NEWS with the web application. 

### 2.4. Endpoint

The primary endpoint was the evaluation of the prognostic capacity of NEWS score in identifying patients with high risk of clinical deterioration in the pre-hospital setting. The secondary outcome was to evaluate the correlation between the NEWS values and the EMS dispatch, and the return priority codes, respectively. Moreover, NEWS score data were compared to emergency department triage evaluation.

### 2.5. Data Collection

The NEWS score was collected with the use of the CovidUP19 web application directly on the field by EMS personnel. The data were stored anonymously on a remote server and included patient age and sex; the NEWS parameters; the date, hours, and minutes of the compilation of the forms; and EMS identification number generated automatically by EMS central database and manually inserted by EMS personnel. To gather all the relevant information for each patient, the NEWS values were associated with the EMS central database and with hospital data by the means of a pseudo-anonymized approach. The data from EMS central database were: EMS ID (this code was used to connect web app data with EMS central database), personal data, ambulance number, dispatch priority code, call location, suspected pathology code, severity return code based on ambulance personnel criteria, outcome of calls, admission emergency department triage code, and hospital outcome. 

### 2.6. Statistical Analysis

Descriptive statistics were used to summarize the available data. Categorical variables are described as count and proportion (%), while continuous variables are expressed as median (quartiles) when not-normally distributed or as mean ± SD when normally distributed. Kolmogorov–Smirnov test was used to assess the parametric distribution. Where applicable, one-way ANOVA or the chi-square test were used to compare values or proportions between groups. Correlations were assessed using Pearson’s or Spearman’s method for normally or non-normally distributed data, respectively. Stepwise multiple linear, logistic binary regression or analysis of covariance (ANCOVA) with Bonferroni correction were applied for multivariate analyses whenever appropriate. NEWS score was used as the main dependent variable. All the analyses were adjusted for possible confounders, including age and gender. Receiving operating curve (ROC) analysis was used to determine the best NEWS value for patient hospitalization; moreover, accuracy, sensitivity, and specificity at that threshold were calculated. All analyses were performed using SPSS version 25 (SPSS Inc., Chicago, IL, USA). The Youden index calculated as (sensitivity + specificity − 1) was used to identify the best cut off [6]. It represents the performance of a diagnostic test: the maximum value of the index is used for selecting the optimum cut-off point. The corresponding positive and negative predicting values were calculated.

### 2.7. Ethics Statement

This study complies with the Declaration of Helsinki, and its protocol was approved by the Azienda USL di Bologna, Maggiore Hospital Institutional Review Board (402-2020-OSS-AUSLBO). Patients’ consent was obtained by EMS crew at arrival on the scene. 

## 3. Results

### 3.1. Descriptive Statistics

The overall characteristics of the included subjects are reported in Table 1. 

**Figure 1:**
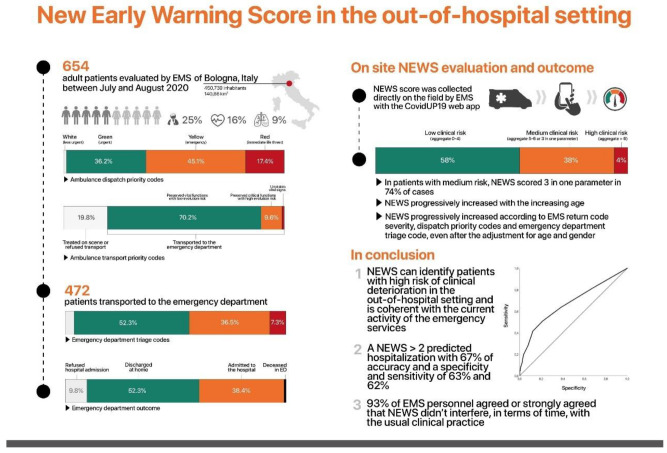
Infographics of the descriptive statistics.

**Figure 2:**
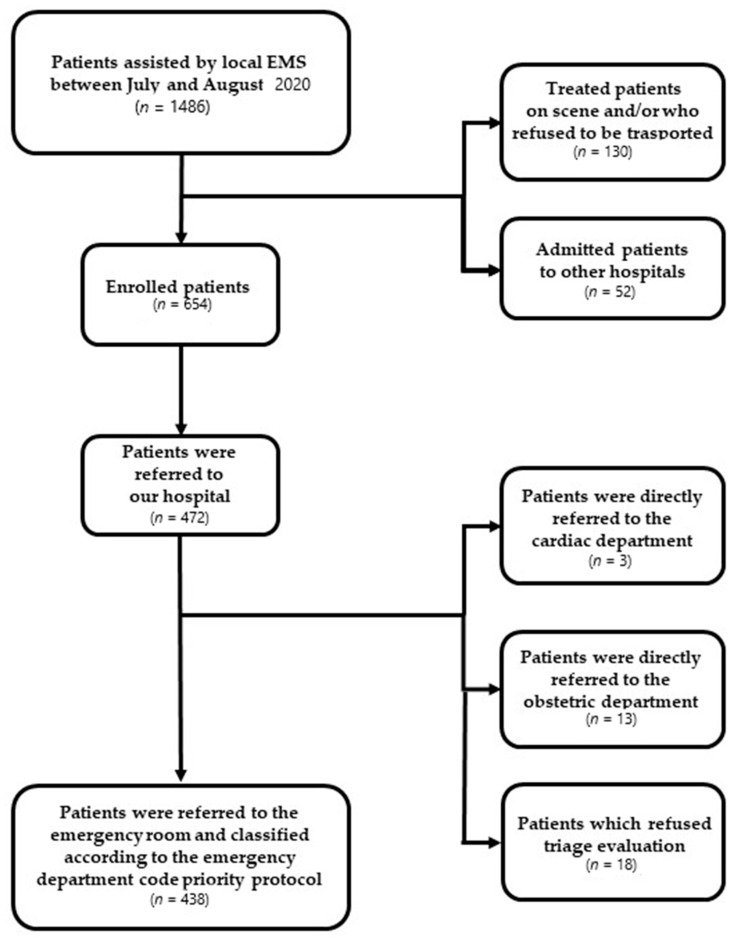
Results flowchart.

Of the 1486 patients assisted from the local EMS during the study period, six hundred and fifty-four were enrolled in the study (44%). The dispatch priority codes for EMS were categorized in four grades (white, green, yellow, red) based on the increasing severity of the patients’ conditions and, in the studied population, were 1.2%, 36.2%, 45.1%, and 17.4%, respectively. The location of calls was mainly from home (72.9%), and the majority of suspected pathology codes were traumatic (25.2%), cardiovascular (16.1%), and respiratory (8.9%) (Table 1). The Maggiore Hospital is the trauma center of Bologna and for this reason, the sample size of the patients included a high percentage of trauma patients. Among the 654 patients evaluated on the field, 130 (19.8%) were treated on scene and/or refused to be transported to the emergency department, while 52 patients were admitted to other hospitals not included in the study. The remaining 472 subjects were referred to our hospital according to the local emergency protocol. Among patients transported to the emergency department, 459 (70.2%) were classified as code 1 (preserved vital functions with low evolution risk and hospitalization with low priority), 63 (9.6%) as code 2 (preserved critical functions with high evolution risk and hospitalization with high priority), and 2 (0.3%) as code 3 (unstable vital signs). Based on traditional in-hospital NEWS thresholds and triggers, the aggregate NEWS value of clinical risk was low (aggregate 0–4) in 558 (58%), medium (aggregate 5–6 or less if at least one individual parameter scored 3) in 207 (38%), and high (aggregate more than 6) in 42 (4%). Indeed, among patients with medium risk, NEWS values scored 3 in a single parameter in 153 (74%) cases, while the aggregate had a value of 5–6 in 54 (26%). In addition, NEWS progressively increased along with the increasing age (r = 0.135; *p* = 0.001). Among the 472 patients admitted to the hospital, 13 and 3 were directly referred to the obstetric or cardiac department, respectively. In addition, 18 refused triage evaluations. The remaining 438 patients were classified according to the emergency department code priority protocol as white (17, 3.9%), green (229, 52.3%), yellow (160, 36.5%), or red (32, 7.3%). Finally, among the 438 patients evaluated at triage, 223 (50.9%) were discharged at home from the ED, 168 (38.4%) admitted to the hospital, 43 (9.8%) refused hospital admission, and 4 (0.9%) died in the emergency department (Figure 1 and Figure 2).

### 3.2. On-Site NEWS Evaluation and Outcome

**Figure 3:**
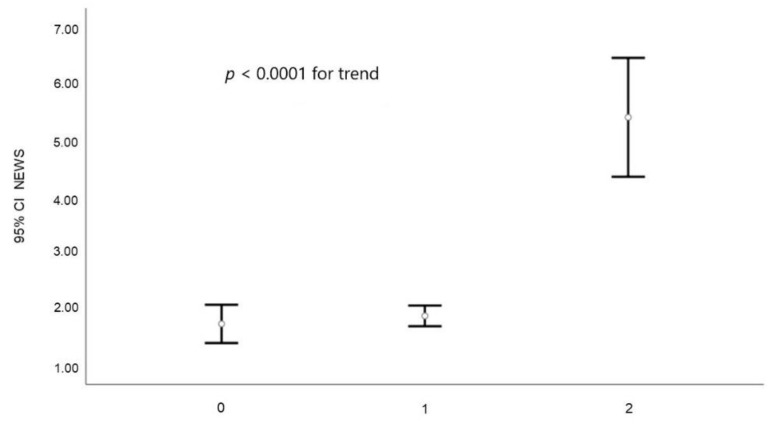
Relation between NEWS value and return code (0 = patient treated on scene or refused the transport, 1 = low severity, 2 = high severity).

NEWS scores were not different between female and male (Table 2); however, it was significantly higher in older subjects when compared to younger (Table 2). Similar to what was observed in the general population, no difference in NEWS score according to the gender was observed in older or younger subjects (1.85 ± 2.09 vs. 1.94 ± 1.99 and 2.64 ± 3.01 vs. 2.42 ± 2.87 for female vs. male in younger and older subjects, respectively; both p = NS). However, some of the items composing the NEWS total score differed according to gender or median age (Table 2). Hence, all the following data were adjusted for sex and gender. At univariate analysis, NEWS values progressively increased according to EMS return-code severity (Figure 3).

The latter association was confirmed even after the adjustment for age and gender (R^2^ = 0.195; *p* < 0.0001). 

**Figure 4:**
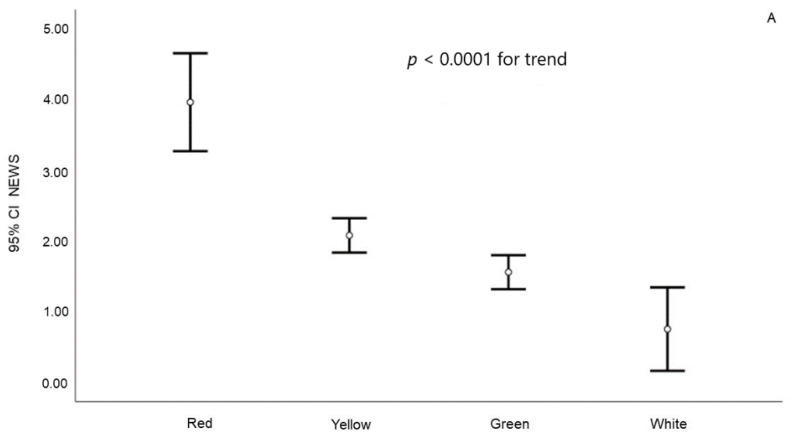

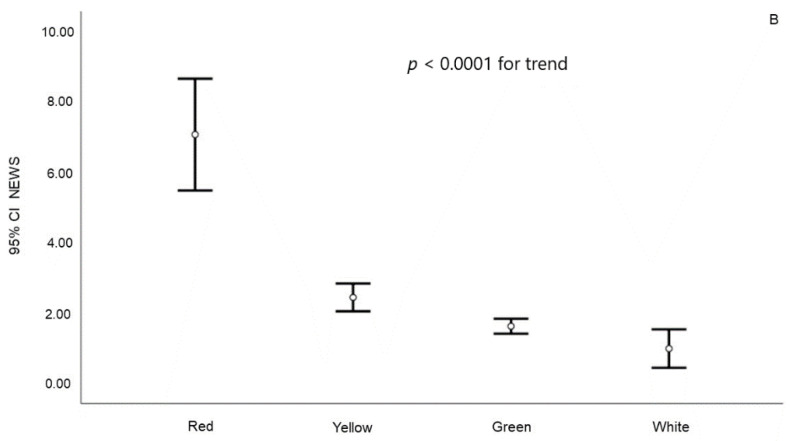
Relation between NEWS value and dispatch priority codes (**A**) and emergency department triage code (**B**).

Similar results were observed when considering dispatch priority codes or those attributed to emergency department triage code (Figure 4A,B). All these differences were confirmed even after the adjustment for age and gender (R^2^ = 0.105 and 0.251, respectively; both *p* < 0.0001). 

**Figure 5:**
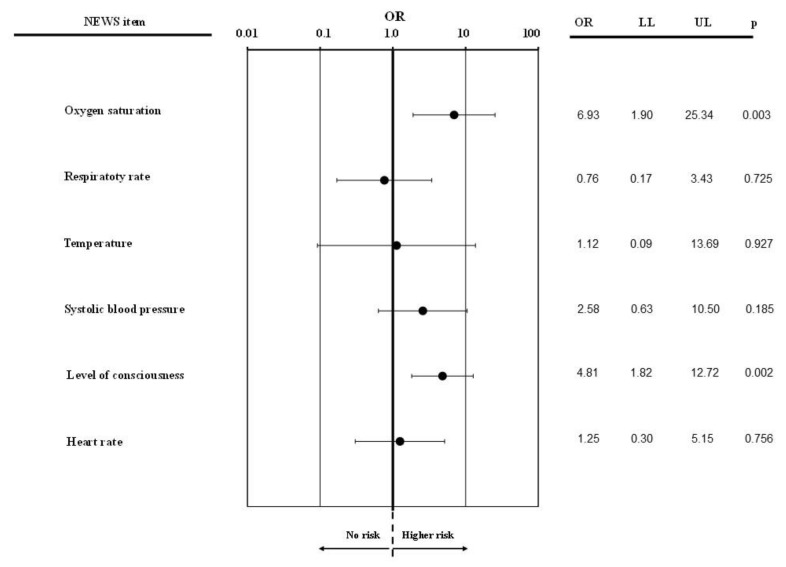
NEWS value with a 3-single parameter and predictors of hospitalization.

**Figure 6:**
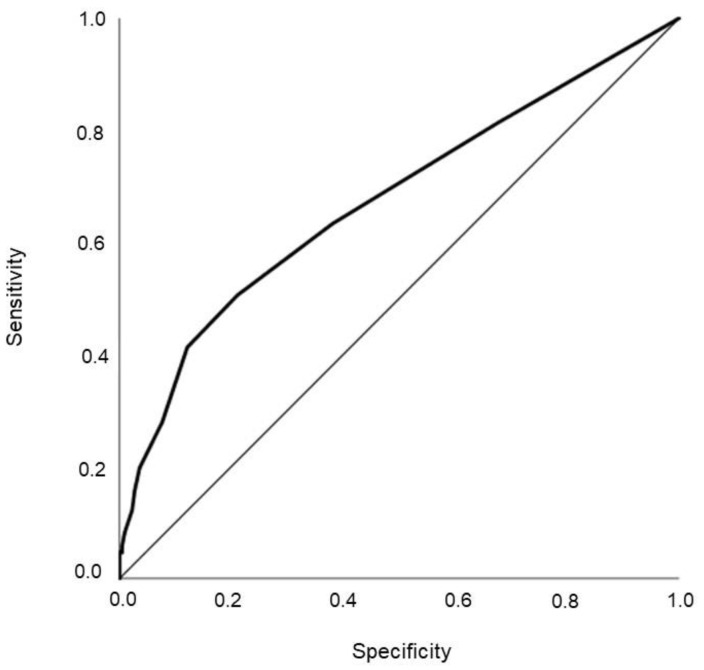
ROC curve analysis and prediction of hospitalization.

Moreover, aggregated value of NEWS was associated with an increased risk of hospitalization even after the adjustment for confounders (OR = 1.30 (1.17; 1.34); *p* < 0.0001). Hence, the risk of hospitalization increased by 30% for each unit increment in the NEWS score. In particular, among NEWS values with a single parameter scoring 3, peripheral oxygen saturation and consciousness evaluation were the best predictors of hospitalization (Figure 5). By applying ROC curve analysis, we found that a NEWS value higher than 2 predicted hospitalization with an accuracy of 67.5 (62.0; 72.9%) ± 0.03% (*p* < 0.0001) and a specificity and sensitivity of 63% and 62%, respectively (Figure 6). Corresponding positive and negative predicting values were 64.9% and negative predictive value of 67.4%; *p* < 0.0001. The Youden Index at that threshold was 0.25 and the corresponding risk ratio 3.83 (2.2.47; 5.94). 

## 4. Discussion

The main finding of this study was that the NEWS could be used in a pre-hospital setting, with acceptable sensitivity and specificity, to estimate the outcome at hospital admission and discharge. Interestingly, peripheral oxygen saturation and consciousness evaluation resulted as the key components of the score that best predicted hospitalization. As anticipated, there was a great deal of evidence proving its efficacy in identifying patients with a high risk of clinical deterioration in in-hospital setting. For instance, a NEWS score > 7 at the time of ICU discharge was a strong independent predictor of clinical deterioration within 24 h [7]. Similar to the in-hospital use, in our pre-hospital setting, a NEWS > 6 or a single NEWS item equal to 3 corresponded to patients with unstable vital signs, and NEWS values progressively increased according to EMS return-code severity. Furthermore, NEWS has proven to be easy to employ as a readily available tool that can be used for predicting in-hospital mortality on all patients admitted to the general ward [8].

This study reports its potential off-label use by EMS to evaluate prehospital patients, facilitating the identification of those needing admission to hospital and procurement of more appropriate care, as other retrospective studies hypothesized [9,10,11,12]. For instance, a retrospective cohort study conducted in West London affirmed that NEWS could be successfully utilized in the pre-hospital emergency setting to predict those patients who were most likely to deteriorate and to thus support the clinicians’ decision-making process [13]. The present study has now showed prospectively the possibility to use NEWS in the pre-hospital setting, demonstrating its utility together with the simplicity of use.

Moreover, NEWS has proved to be useful to detect early sepsis in pre-hospital emergency care, confirmed by a study carried out in UK that demonstrated how NEWS in prehospital care was associated with improved outcomes in patients with suspicion of sepsis [14]. In our cohort, only 1.1% of patients were at risk for infection/sepsis, while the majority had a traumatic or a cardiovascular injury.

Nowadays, there are several scales used by the emergency crew to assess the criticality of patients, such as the Glasgow coma scale (GCS), the revised trauma score (RTS), the quick sequential organ failure assessment (qSOFA). All of them, without any doubts, are the basis for patient assessment, but, on the other hand, there is evidence that NEWS is superior in a pre-hospital environment at identifying patients at risk of adverse outcomes, in particular, in comparison with qSOFA [11].To the best of our knowledge, this study is one of the few prospective, observational cohort studies that evaluated the use of NEWS in pre-hospital settings to assess the predictive value of vital parameters measurement by an ambulance crew directly on the field. In this study, as in similar observational retrospective studies, we enrolled a significant sample of pre-hospital patients, and we described the prevalence and the distribution of NEWS in a representative population of emergency calls received from our dispatch center [9,10,11,12]. Interestingly, our results show that, in most of the pre-hospital settings, high scores are reasonably uncommon, and NEWS scores in a pre-hospital setting are much lower than those calculated inside the hospital [8]. Furthermore, the results highlighted the coherence of NEWS with the current organizational activity of the urgent and emergency-care services. This is deduced by several correlations. First, NEWS increased as a function of the given dispatch priority code following the emergency calls; secondly, the same relation was observed according to EMS return code; and, finally, it increased with the increase of the emergency department triage code. These links seem to support the capability of NEWS in identifying patients with high risk of clinical deterioration even in the pre-hospital setting and to predict the subsequent evolution of severity assessment from the dispatch priority to the pre-hospital-care phase and finally to hospital triage. We also tested the capability to use web-app technology to get an immediate score-minimizing time factor, which plays a vital role in the urgent and emergency care set. NEWS sees its greatest application in all cases in which specific factors that explicitly highlight the critical clinical condition of the patient are absent, not to overlook potential signs of critical disease or severe damage that may not be intercepted in initial evaluation [15]. At the same time, it should not be used as a substitute of critical thinking but as an integrative and supportive tool for ambulance crews in the assessment of the patients. 

The main limitations of this study lie in its monocentric design and in the limited sampling of potentially recruitable cases (44%). This was mainly due to the dispatch of ambulances not involved in the present study, while less than 1% of cases was due to missed data collection from the attending ambulance crew. Another concern is not having calculated the sample size; however, this was a preliminary study aiming to evaluate prospectively the feasibility of the proposed approach to the pre-hospital critical patient. Hence, further studies are advisable in order to validate the score. Nevertheless, the collected sample is sufficiently representative of the average distribution of the dispatch priority codes sent by our dispatch center. In any case, it should be recognized that the validity of NEWS in non-hospital settings should be confirmed by other studies before drawing final conclusions. On the other hand, the main strengths of this study are its prospective observational design and the implementation of data collection tools in order to minimize the risk of interfering with clinical practice (Appendix C). Further studies will be needed to confirm these results and to evaluate the application of NEWS in specific sub-settings, such as ALS teams’ activation.

## 5. Conclusions

This prospective, observational study showed that the use of NEWS in urgent and emergency-care services is promising, as it does not affect operation time; moreover, it creates a direct and standardized language between the different figures operating in the pre-hospital setting (nurses, doctors, and rescuers) and might assist decision making in terms of severity-code assignment and resource utilization. The potential implementation should be validated in a wider network of critical patients that connects the out-of-hospital emergency with the intra-hospital setting.

## Figures and Tables

**Table 1:** Overall characteristics of the included subjects.

EMS Dispatch Priority Codes	Patients (%)	Emergency Department Triage Code	Patients (%)
White	8 (1.2%)	White	17 (3.9%)
Green	237 (36.2%)	Green	229 (52.3%)
Yellow	295 (45.1%)	Yellow	160 (36.5%)
Red	114 (17.4%)	Red	32 (7.3%)
Total	654	Total	438
Place of calls	Patients (%)	Emergency Department Outcome	Patients (%)
Residence	477 (72.9%)	Discharged at home	223 (50.9%)
Work/Office	18 (2.8%)	Admitted to the hospital	168 (38.4%)
Public building	15 (2.3%)	Refused hospital admission	43 (9.8%)
Street	101 (15.4%)	Deceased in ED	4 (0.9%)
Sport facilities	2 (0.3%)	Total	438
Other	41 (6.3%)		
Total	654		
Suspected pathology code	Patients (%)	Return code	Patients (%)
Traumatic	165 (25.2%)	0	130 (19.9%)
Cardiovascular	105 (16.1%)	1	459 (70.2%)
Respiratory	58 (8.9%)	2	63 (9.6%)
Neurologic	51 (7.8%)	3	2 (0.3%)
Psychiatric	27 (4.1%)		
Neoplastic	7 (1.1%)		
Intoxication	17 (2.6%)		
Metabolic	6 (0.9%)		
Gastroenteric	48 (7.3%)		
Urologic	10 (3.7%)		
Ophthalmic	1 (0.2%)		
ENT	4 (0.6%)		
Obstetric	16 (2.4%)		
Infectious	7 (1.1%)		
Other pathologies	115 (17.6%)		
Not identified	3 (0.5%)		
Total	654		

**Table 2:** NEWS total score and sub-domain score according to population median age or gender.

	Female	Male	*p*	<68 Years Old	≥68 Years Old	*p*
Respiratory rate	0.29 ± 0.79	0.24 ± 0.72	0.413	0.26 ± 0.74	0.27 ± 0.77	0.775
Oxygen saturation	0.43 ± 0.86	0.34 ± 0.76	0.161	0.20 ± 0.61	0.57 ± 0.94	0.000
Temperature	0.23 ± 0.55	0.26 ± 0.56	0.501	0.23 ± 0.53	0.26 ± 0.58	0.551
Systolic Pressure	0.39 ± 0.74	0.52 ± 0.80	0.038	0.44 ± 0.71	0.48 ± 0.84	0.483
Heart rate	0.54 ± 0.75	0.53 ± 0.78	0.803	0.62 ± 0.79	0.45 ± 0.73	0.006
Consciousness	0.20 ± 0.75	0.20 ± 0.76	0.969	0.10 ± 0.54	0.30 ± 0.91	0.001
Total score	2.19 ± 2.51	2.21 ± 2.58	0.936	1.89 ± 2.05	2.51 ± 2.93	0.002

**Figure A1:**
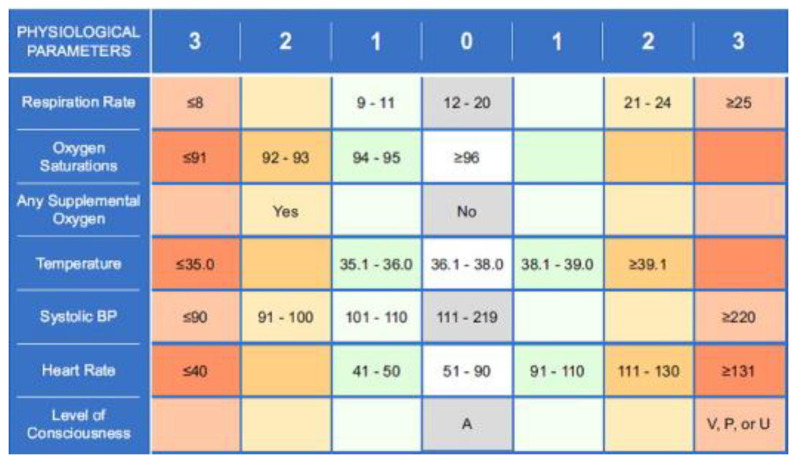
National Early Warning Score (Royal College of Physicians, 2012).

**Figure A2:**
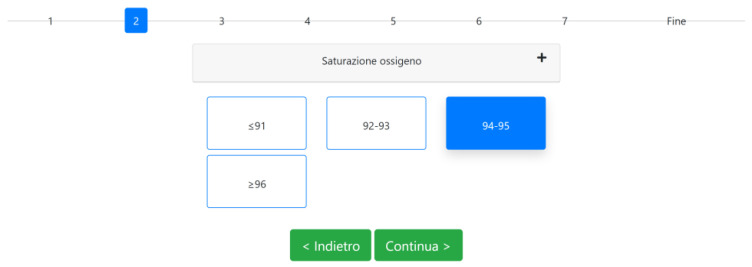
CovidUP19 Web-App Screenshot.

**Figure A3:**
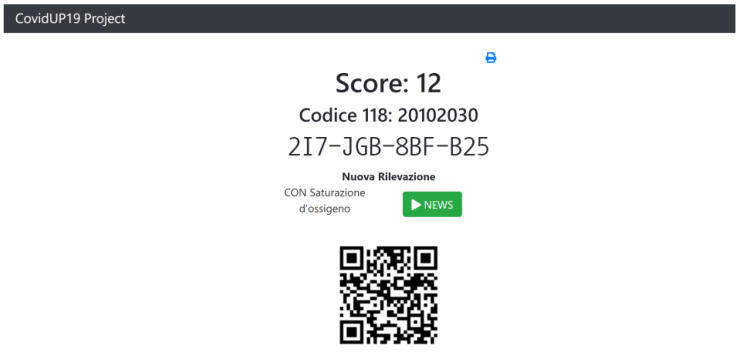
Descriptive Analysis of the EMS Questionnaires about the Use of NEWS.

## Data Availability

Internal EMS and Maggiore Hospital digital database is not available as public archive.

## Appendix C

A crew personnel questionnaire was designed to collect feedback about the use of NEWS. Each participant was asked to complete the questionnaire based on a seven-point Likert scale (LS) designed to explore the applicability on the field, potential implementation as a tool to measure the clinical situation at scene, and time spent in calculating the score. The questionnaire data were collected anonymously with a JotForm form (JotForm, San Francisco, CA, USA) developed for the study. Forty-six EMS personnel out of sixty-nine (67%) involved in the study filled out the whole questionnaire; among them, the majority (80%) were BLS-skilled rescuers, while the remaining 20% were nurses attending the ILS vehicles. Among the responders, 48% recruited more than twenty cases with no mention of any kind of difficulties (98%). Since the majority of the responders (74%) had never used the NEWS before, we investigated whether the initial training phase had been exhaustive enough in providing all the necessary contents to use NEWS properly: a total of 54% and 41%, respectively, agreed and strongly agreed, according to the proposed Likert scale. Moreover, the developed web application turned out to be clear, comprehensible, and easy-to-use for 50% and 39% of the responders, which, respectively, strongly agreed and agreed with the proposed statements. Furthermore, 54% agreed and 39% strongly agreed that NEWS did not interfere with the usual clinical practice in terms of time; only 4% and 2%, respectively, partially agreed and were uncertain. Concerning its usefulness, 17% and 50% of answers agreed and strongly agreed, respectively, in stating that NEWS could be a potentially useful and relevant tool to assess patients in out-of-hospital settings. Approximately half of the EMS personnel who completed the questionnaire considered the use of NEWS as a valid supplementary tool to recruit ALS vehicles in those critical cases in which advanced life-support skills are required; an additional tool to support ambulance personnel in decision making about the severity of the return code; and an integrative tool to enhance, in terms of time and suitability, the admission to the emergency department triage. Lastly, we asked whether participants would deploy the score into emergency out-of-hospital clinical practice. The results were characterized by heterogeneity, since 22% answered absolutely yes, 39% yes, 22% probably yes, and 11% probably no. From the results, NEWS application was not perceived as an obstacle in terms of time and operativity during the management of urgent and emergency situations. In fact, as 54% agreed and 39% strongly agreed that NEWS did not interfere with the usual clinical practice in terms of time but rather was a valid adjunct to the clinical decision for urgent care and/or hospital admission needs.

## References

[1] Smith G.B., Prytherch D.R., Schmidt P.E., Featherstone P.I. (2008). Review and performance evaluation of aggregate weighted ‘track and trigger’ systems. Resuscitation.

[2] Yeung J., Scapigliati A., Hsieh M., Boulton A., Saviani M., Georgiou M., Schnaubelt S., Bray J., Bhanji F., Bigham B. Rapid Response Systems in Adults Consensus on Science with Treatment Recommendations [Internet] Brussels, Belgium: International Liaison Committee on Resuscitation (ILCOR) Education, Implementation and Teams Task Force. https://costr.ilcor.org/document/rapid-response-systems-in-adults-systematic-review.

[3] Smith G.B., Prytherch D.R., Meredith P., Schmidt P.E., Featherstone P.I. (2013). The ability of the National Early Warning Score (NEWS) to discriminate patients at risk of early cardiac arrest, unanticipated intensive care unit admission, and death. Resuscitation.

[4] New National Early Warning Score Could Save 6000 Lives. https://www.rcplondon.ac.uk/news/new-national-early-warning-score-could-save-6000-lives.

[5] Semeraro F., Scquizzato T., Scapigliati A., Ristagno G., Gamberini L., Tartaglione M., Dell’Arciprete O., Mora F., Cordenons F., Del Giudice D. (2020). New Early Warning Score: Off-label approach for Covid-19 outbreak patient deterioration in the community. Resuscitation.

[6] Schisterman E.F., Perkins N.J., Liu A., Bondell H. (2005). Optimal Cut-point and Its Corresponding Youden Index to Discriminate Individuals Using Pooled Blood Samples. Epidemiology.

[7] Uppanisakorn S., Bhurayanontachai R., Boonyarat J., Kaewpradit J. (2018). National Early Warning Score (NEWS) at ICU discharge can predict early clinical deterioration after ICU transfer. J. Crit. Care.

[8] Lee Y.S., Choi J.W., Park Y.H., Chung C., Park D.I., Lee J.E., Lee H.S., Moon J.Y. (2018). Evaluation of the efficacy of the National Early Warning Score in predicting in-hospital mortality via the risk stratification. J. Crit. Care.

[9] Silcock D.J., Corfield A.R., Gowens P.A., Rooney K.D. (2015). Validation of the National Early Warning Score in the prehospital setting. Resuscitation.

[10] Pirneskoski J., Kuisma M., Olkkola K.T., Nurmi J. (2019). Prehospital National Early Warning Score predicts early mortality. Acta Anaesthesiol. Scand..

[11] Hoikka M., Silfvast T., Ala-Kokko T.I. (2018). Does the prehospital National Early Warning Score predict the short-term mortality of unselected emergency patients?. Scand. J. Trauma. Resusc. Emerg. Med..

[12] Silcock D.J., Corfield A.R., Staines H., Rooney K.D. (2019). Superior performance of National Early Warning Score compared with quick Sepsis-related Organ Failure Assessment Score in predicting adverse outcomes: A retrospective observational study of patients in the prehospital setting. Eur. J. Emerg. Med..

[13] Shaw J., Fothergill R., Clark S., Moore F. (2017). Can the prehospital National Early Warning Score identify patients most at risk from subsequent deterioration?. Emerg. Med. J..

[14] Pullyblank A., Tavaré A., Little H., Redfern E., Le Roux H., Inada-Kim M., Cheema K., Cook A. (2020). Implementation of the National Early Warning Score in patients with suspicion of sepsis: Evaluation of a system-wide quality improvement project. Br. J. Gen. Pr..

[15] Williams T.A., Tohira H., Finn J., Perkins G.D., Ho K.M. (2016). The ability of early warning scores (EWS) to detect critical illness in the prehospital setting: A systematic review. Resuscitation.

